# P236: Effect of a prevention campaign on the prevalence of infections among patients in Belgian psychiatric hospitals: a dynamic prospective cohort study (2001-2010)

**DOI:** 10.1186/2047-2994-2-S1-P236

**Published:** 2013-06-20

**Authors:** R Haenen, S Vandenbroeck, G Moens, A De Schrijver, K Johannik, L Godderis

**Affiliations:** 1 IDEWE, external service for prevention and protection at work, Heverlee, Belgium; 2 Occupational, Environmental and Insurance Medicine, KU Leuven, Leuven, Belgium; 3 Epidemiology and Social Medicine, UA, Antwerpen, Belgium

## Introduction

Infection control programs are crucial in reducing healthcare associated infections and their inherent costs. Few data on infection prevalence in psychiatry and effectiveness of prevention are available.

## Objectives

In this study, we investigated in psychiatric institutions the evolution of 1) point prevalence of infections, infected patients, and antibiotics’ use; 2) prevalence infection rates before and after a hand hygiene campaign.

## Methods

Demographics, antibiotics’ use, presence and type of infections were registered by an assessor using a standardized form. The criteria to determine the presence of an infection were based on the criteria of the Centers for Disease Control (1988).

## Results

The overall infection, resp. infected patients prevalence was 18.9% and 16.5%. The three most frequent infections were 1) skin or soft tissue (38.7%), 2) lower (22.5%) and 3) upper respiratory tract infections (11.4%). The prevalence of antibiotics’ use was 2,7%. The implementation of a hand hygiene campaign resulted in significant decreased prevalence of infected patients: 17.7% (95% CI: 17.1-18.3) before versus 15.1% (95% CI: 14.4-15.7) in the 4-years after the implementation. Antibiotics’ use among infected patients diminished from 17.5% to 14.0% (p<0.001).

## Conclusion

These results are suggestive for a statistically and clinically significant effect of hand hygiene campaigns. Per 1000 patients/year, 37 infections and 26 infected patients have potentially been avoided.

## Disclosure of interest

None declared

